# A Pilot Clinical Study Assessing Treatment of Canine Hip Dysplasia Using Autologous Protein Solution

**DOI:** 10.3389/fvets.2019.00243

**Published:** 2019-08-09

**Authors:** Samuel P. Franklin

**Affiliations:** Colorado Canine Orthopedics and Rehab, Colorado Springs, CO, United States

**Keywords:** autologous protein solution, APS, canine, hip dysplasia, osteoarthritis

## Abstract

Five dogs with bilateral hip dysplasia and without osteoarthritis of other joints were enrolled in this pilot study. Objective kinetic data using a pressure sensitive mat and owner assessments using the canine brief pain inventory (CBPI) and Liverpool Osteoarthritis for Dogs (LOAD) questionnaires were obtained prior to treatment. Enrolled dogs were treated in one hip with autologous protein solution (APS) and the contralateral hip was injected with an equal volume of saline. The hip to be treated was selected using a random number generator. At exactly 28 days following treatment dogs were re-assessed using the pressure sensitive mat and the CBPI and LOAD questionnaires. No dogs were treated with any other medications or supplements throughout the study period. Assessment of the total pressure index (TPI) collected using the pressure sensitive mat showed that the hips treated with APS improved significantly more than hips treated with saline (*p* = 0.0005) and that the hips treated with APS bore significantly more weight than the hips treated with saline at day 28 (*p* < 0.05). Statistically significant improvement was noted by owners in “pain” and “function” as assessed by the CBPI as well “mobility at exercise” using the LOAD questionnaire. This pilot study provided proof of principle that APS is beneficial in treating pain and lameness in dogs affected by coxofemoral osteoarthritis.

## Introduction

Autologous protein solution (APS) is an autologous blood product that is prepared patient-side and is purported to have anti-inflammatory cytokines, such as interleukin receptor-1 antagonist, and anabolic proteins that could be beneficial in the treatment of osteoarthritis (1). Previous studies in horses (1) and dogs (2), provide initial evidence that intra-articular administration of APS can be effective in reducing pain and lameness in these species when compared to intra-articular saline controls. While the previous studies were done well and provide evidence of benefit, these studies had some minor limitations. The study performed in dogs (2) did not include a uniform population of patients with regard to the affected joints, the underlying pathology, or whether surgery had been performed previously. Furthermore, none of the dogs in that study had hip osteoarthritis. Consequently, we planned on performing a large study in a uniform population of patients comparing APS to control for treating hip osteoarthritis in dogs. However, prior to initiating a large, lengthy, and costly study, we sought to perform a pilot study in a limited number of dogs to evaluate study methodology and feasibility, as well as to facilitate performing a power analysis for a larger study.

The specific objectives of this pilot study were to: (1) evaluate the likelihood and ease of enrolling study subjects meeting the study inclusion criteria, (2) evaluate the methods including treating one hip in dogs with APS and the contralateral hip with a saline control and using a pressure sensitive mat as the primary outcome variable, (3) enable performance of a power analysis to determine how many dogs would need to be enrolled in a study that was adequately powered to detect a clinically-relevant difference between APS and a saline control.

## Methods and Materials

### Enrollment Criteria and Screening

With approval by the Animal Care and Use Committee of Colorado Canine Orthopedics and Rehab, we sought to enroll 5 dogs between 22 and 55 kg in body weight and between 2 and 10 years of age with bilateral hip osteoarthritis and without osteoarthritis of their shoulders, elbows, or knees. All dogs were required to have no other medical conditions and to be off all medications and supplements during the course of the study. Numerous dogs were pre-screened remotely and twelve dogs were evaluated in-house for potential enrollment. As part of that screening process the principal investigator consulted with the owner to discuss history, medical problems, and medications. In addition, a physical examination and subjective gait assessment were performed by the principal investigator, and radiographs were made of the hips, stifles, shoulders, and elbows. Specifically, medial-lateral radiographs were made of the shoulders, elbows, and stifles, and a lateral view of the pelvis was made. A cranial-caudal radiograph was made of both elbows simultaneously and a ventro-dorsal radiograph of the pelvis that included both stifles in the frontal plane were made. Any radiographic or physical exam evidence of pathology of any joints other than the coxofemoral joints resulted in exclusion of the patient from the study. Asymmetry in gait based upon the investigator's subjective gait assessment or the owner's history resulted in exclusion as the goal was to enroll relatively symmetrically affected dogs.

### Baseline Data Collection

Following screening, dogs that met the inclusion criteria were enrolled and all owners provided written, informed consent for inclusion in the study. Prior to treatment owners then completed the Canine Brief Pain Inventory (CBPI) and the Liverpool Osteoarthritis in Dogs (LOAD) questionnaires. Dogs were also trotted across a pressure sensitive mat (Gait4Dog®, CIR Systems, Franklin, NJ) to collect objective gait data. We sought to obtain a minimum of 10 valid trials across the mat. A valid trial was considered one in which the leash was slack and not influencing the gait, the dog was trotting in a straight line while looking straight ahead without turning its head, and in which the dog maintained a relatively consistent pace. Dogs were typically trotted 20–40 times across the mat in order to ensure obtaining 10 acceptable trials. All trials were video-recorded for subsequent review.

### APS Treatment

After collection of baseline data dogs were sedated with 5 μg/kg of Dexmedetomidine and 0.2 mg/kg of Butorphanol administered intravenously. An area over the jugular vein was then clipped and aseptically prepared and an 18-gauge 2″ IV catheter was then placed in the jugular vein. A 60 ml syringe that was pre-loaded with 5 ml of ACD-A was then attached to the catheter and filled with blood to its full 60 ml volume. The syringe was then gently inverted multiple times to mix the blood and anti-coagulant. A small volume (0.5–1 ml) of such blood was placed in a small purple top tube with EDTA for performing a whole blood complete blood count (CBC) on an in-house blood analyzer (Element HT5, Heska, Loveland, CO). The remaining blood was then used to prepare APS using the Pro-Stride® APS kit according to manufacturer instructions. Following APS preparation 1 ml of APS was collected for administration to the patient and ~0.5–1 ml was taken for a CBC on the APS.

One hip was then aseptically prepared for injection of saline and one hip was aseptically prepared for injection of APS. The treatment side was randomized *a priori* by the patient number using a random number generator. Prior to injection of either APS or saline (1 ml of each) in the respective side, joint fluid was successfully aspirated from all hips to confirm intra-articular delivery of the saline or APS. Following treatment dogs were reversed from their sedation using Atipamezole (equal volume as the Dexmedetomidine; given intramuscularly) and discharged to the owners. Owners were provided with instructions on how to monitor for adverse events and a daily log into which they were supposed to report any adverse events or use of any medications or supplements. Owners were informed that they were allowed to use medications or supplements if necessary but were requested to contact the principal investigator prior to initiating using of any medications or supplements. All dogs were allowed to resume activity without limitation.

### Post Treatment Data Collection

Exactly 28 days following treatment dogs were re-evaluated in-house. The principal investigator consulted with the owners, reviewed the daily log with them, and recorded information regarding any adverse events and use of medications. Owners then repeated the CBPI and LOAD questionnaires without having access to the initial CBPI and LOAD questionnaires they had completed 28 days earlier. The same owner completed the CBPI and LOAD questionnaires at both time points. Dogs were then trotted across the pressure sensitive mat again just as they had been prior to treatment with APS.

## Data Evaluation and Statistical Analyses

### Radiography

Radiographs of the hips were evaluated by a board-certified radiologist blinded to the study design and treatment allocation. Hips were graded using the Orthopedic Foundation for Animals grading classifications dogs with hip dysplasia.

### Objective Kinetic Data

All trials of dogs trotting across the pressure sensitive mat, both before treatment and 28 days post treatment, were reviewed. Such review included reviewing the video of them trotting across the mat. If the trial met the aforementioned criteria for a “valid” trial, the trial was then quantitatively evaluated to obtain information on the relative weight bearing of each limb. Only those trials in which there was a minimum of 2 full gait cycles and <10% variation in the dog's velocity were included. The pressure sensitive mat did not record or report the instantaneous velocity. The lowest number of valid trials obtained and used in statistical analyses for any dog was 11 and the maximum number of valid trials obtained was 18.

Once the gait data were quantified the total pressure index (TPI) of the two pelvic limbs were exported for assessment. The TPI was selected as the outcome variable of interest before the study was conducted or the data analyzed (i.e., *a priori*). These data were then evaluated using a linear mixed model (LMM). The LMM included fixed factors for treatment and the time of the assessment (i.e., pre vs. post treatment) and a treatment by time (i.e., pre/post) interaction effect. The LMM also included random intercepts for each dog and each limb to account for within dog and within limb correlations. Satterthwaite degrees of freedom method was used. Multiple comparisons were adjusted for with Tukey's test. Model residuals were examined to evaluate the assumption of normality. These analyses were performed using SAS V 9.4 (Cary, NC).

### CBPI

The CBPI consists of 4 questions in which the owner assesses the dog's pain and six questions that evaluate the dog's function. For each of these questions, owners can provide an answer from 0 to 10 with 0 being consistent with a normal dog and 10 being consistent with either more pain or decreased function. The total scores for the first 4 questions (i.e., pain) were summed for each dog both prior to and following treatment. Similarly, the total scores for the 6 questions assessing function were summed for each dog both prior to and following treatment. Finally, there was one last question assessing “overall impression” that could be scored poor, fair, good, very good, or excellent by the owner. We changed these responses to this question to an ordinal scale (1–5) with 1 being poor and 5 being excellent. For each of these three parts of the CBPI (pain, function, and overall impression) a paired *T*-test was performed to evaluate whether there was a significant improvement as determined by the owner. The *T*-tests were two-tailed with an alpha value specified *a priori* as 0.05.

### LOAD

The LOAD has 5 questions that assess “mobility generally” and 8 questions that assess “mobility at exercise.” Each of these 5 questions allowed 5 discrete responses by the owner. For example, for the answer to the question regarding general mobility the owners could select very good, good, fair, poor, or very poor. We converted the responses for all questions into an ordinal scale (1–5) with higher scores consistent with decreased mobility. As for the CBPI, the total score for the 5 questions assessing “general mobility” were summed for each dog both prior to and following treatment. The 8 questions assessing “mobility at exercise” were similarly summed for each dog both prior to and following treatment. For each of these two parts of the LOAD (“general mobility” and “mobility at exercise”) a paired *T*-test was performed to evaluate whether there was a significant improvement as determined by the owner. The *T*-tests were two-tailed with an alpha value specified *a priori* as 0.05.

## Results

### Patient Demographics

Of the twelve dogs screened for possible inclusion, five met the inclusion criteria and were enrolled. Note that one dog was excluded because although the dog met all inclusion criteria with regard to radiography, joints affected, and history, the dog was visibly asymmetrical and predominantly lame on one pelvic limb based upon subjective gait evaluation. The 5 dogs included 3 golden retrievers, 1 German shepherd dog, and 1 shepherd mixed breed. Three dogs were spayed females while two dogs were castrated male dogs. The mean weight was 82.6 lbs (range 61.6–96.8). The mean age was 4.9 years (range 3.2–8.2).

**Figure 1:**
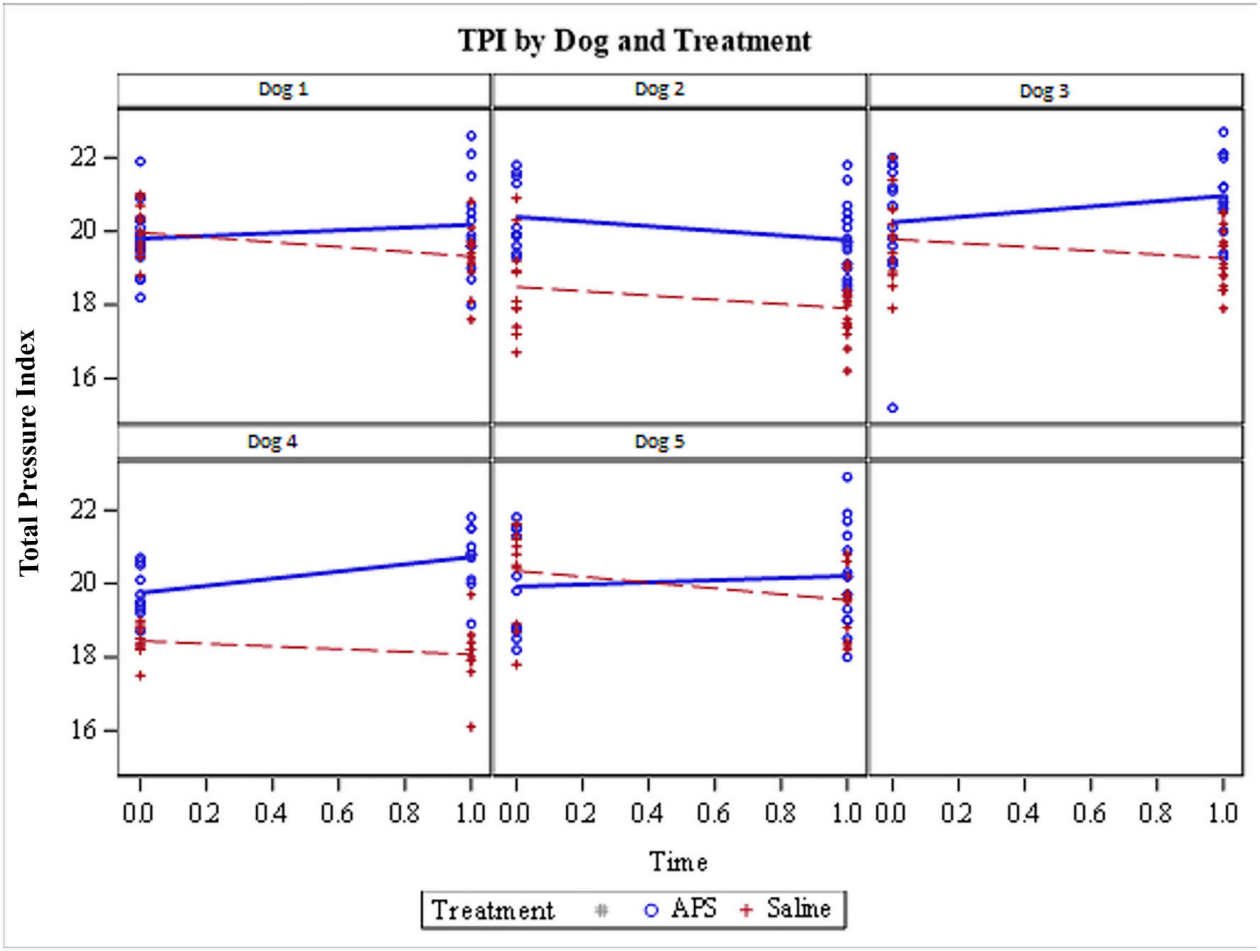
Total Pressure Index (TPI) for each of the 5 dogs included in the pilot study. Limbs treated with APS are represented by blue data points (and lines) and the limbs treated with saline are shown by red data points (and lines). TPI was measured at two time points (day 0 immediately prior to treatment and day 28 following treatment). Four out of 5 dogs showed improvement in TPI in the APS-treated limb; the one exception was Dog 2. All 5 dogs showed a decrease in the relative pressure distribution to the saline-treated limbs.

Both hips of three dogs were classified as having severe hip dysplasia. In one dog both hips were classified as having moderate hip dysplasia. In one dog (dog 4; see Figure 1) one hip was classified as having severe hip dysplasia and the other has having moderate hip dysplasia. In this dog, the hip that had moderate dysplasia was subsequently randomly allocated to treatment with APS while the radiographically severely affected hip was treated with saline.

### Medication Usage

All dogs were off all medications and supplements a minimum of 1 week prior to the start of the study. All dogs remained free of all medications and supplements for the duration of the 28-day study.

### Adverse Events

One dog was sore as determined by the owner for the first 48–72 h following injection. By 72 h following injection the owner thought the dog was more comfortable and functional than prior to injection. One other dog had pruritus at the injection site and would lick or chew at the area. This started the day following injection and lasted for 3 days. Neither dog obtained any treatment for either adverse event.

### Complete Blood Count Data

The results of the CBCs for each dog were relatively consistent in showing that production of APS reduced concentrations of red blood cells and platelets while increasing the concentrations of all leukocyte types. Red blood cells concentrations were decreased on average by 80%, platelet concentrations were reduced by an average of 20%, and leukocyte concentrations were increased on average by 11-fold in the APS. These data are shown in Table 1.

**Table 1:** Cellular composition of the whole blood and APS.

**Cell type**	**WBC**	**NEU**	**LYM**	**MONO**	**EOS**	**BAS**	**RBC**	**PLT**
Units	^*^10^3^/μL	^*^10^3^/μL	^*^10^3^/μL	^*^10^3^/μL	^*^10^3^/μL	^*^10^3^/μL	^*^10^6^/μL	^*^10^3^/μL
Mean (whole blood)	8.7	6.4	1.7	0.3	0.3	0.006	6.8	208
Mean (APS)	94.7	67.7	20.2	3.5	3.2	0.09	1.2	148
Mean fold increase	11.3	11.2	11.7	14.2	10.6	~15	0.2	0.8
Stdev fold increase	3.3	4.0	4.4	4.6	4.6	NA*	0.1	0.5

*WBC, white blood cells; NEU, neutrophils; LYM, lymphocytes; MONO, monocytes; EOS, eosinophils; BAS, basophils; RBC, red blood cells; PLT, Platelets. Stdev, standard deviation. Four of 5 whole blood samples were completely devoid of basophils (i.e., 0/microliter), making calculation and estimation of fold increase problematic*.

### Owner Assessment Data

#### CBPI

The mean CBPI “pain” score prior to treatment was 9.4 (standard deviation ±6.2) and following treatment was 1.8 (±2.9), consistent with improvement in pain. The improvement was statistically significant (*p* = 0.03). The mean CBPI “function” score prior to treatment was 13.2 (±10.5) and the mean function score following treatment was 2.8 (±4.1), also consistent with improvement. This improvement was statistically significant (*p* = 0.047). The mean “overall impression” score prior to treatment was 3.2 (±0.45); the mean following treatment was 3.6 (±0.55). This improvement was not statistically significant (*p* = 0.09).

### LOAD

The mean score for “general mobility” prior to treatment was 12.6 (±2.7) and following treatment the score was 10.4 (±1.7). This difference was not statistically significant (*p* = 0.09). The mean score for “mobility at exercise” was 20.3 (±4.97) prior to treatment and was 17.1 (±3.94) following treatment. This improvement was statistically significant (*p* = 0.02).

### Total Pressure Index

TPI was not significantly different between limbs pre-treatment (*p* = 0.49). TPI was significantly higher in limbs treated with APS than limbs treated with saline on day 28 by an average of 1.5 (95% confidence interval 0.1–3.0; *p* = 0.0419). TPI increased in APS-treated limbs by an average of 0.3 (95% confidence interval of −0.15 to 0.80; *p* = 0.2927). TPI decreased on average by 0.60 (95% confidence interval −1.07 to −0.12; *p* = 0.007) in saline-treated limbs. The change in TPI from pre-treatment values was significantly greater in the APS-treated limbs than in the saline-treated limbs (*p* = 0.0005). The mean TPIs for each limb (saline-treated vs. APS-treated) and at each time point (days 0 and 28) are shown in Table 2.

**Table 2:** Total pressure index.

**Treatment**	**Day**	**TPI (%)**	**Standard error**
Saline	0	19.4129^a^	0.2898
APS	0	20.0089^a, b^	0.2898
Saline	28	18.8177^c^	0.2863
APS	28	20.3317^b^	0.2863

*The TPI reflects the relative distribution of weight borne by a limb, out of all 4 limbs. If the two estimates of TPI share a common letter they are not statistically significantly different*.

If one looks at each dog individually, the TPI decreased for the saline-treated limb over the 28-day study period for all 5 dogs (Figure 1). Conversely, the TPI increased for the APS-treated limb in 4 out of 5 patients. The TPI was higher on day 0 prior to treatment for the APS-treated limb in 3 dogs and the TPI was higher on day 0 (prior to treatment) for the saline-treated limb in 2 dogs (Figure 1).

## Discussion

This was a pilot study with few dogs and primary objectives of assessing the ease of enrolling study subjects and the study methodology as well as enabling performance of a power analysis for guiding design of future studies using such methodology. This pilot study showed that the majority of dogs screened were not candidates for inclusion. Dogs were commonly excluded because they had palpable or radiographic pathology of other joints or had a subjectively asymmetrical gait. Despite excluding the majority of dogs for these reasons, sufficient dogs were identified to meet the desired enrollment of 5 dogs and we conclude that larger studies using the same enrollment criteria are feasible. Furthermore, the study showed that the study design and methodology were sufficient to enable identification of a statistically significant difference between the two different treatments using objective kinetic data. Thus, we conclude that this study design and methodology are appropriate for use in future studies evaluating the use of APS in dogs with hip osteoarthritis. Our third objective of gaining sufficient data to perform a power analysis was rendered irrelevant because the pilot study demonstrated that APS resulted in a statistically significant better outcome when compared to the sham (saline) control.

The data show that four out of the 5 dogs had an increase in TPI on the saline treated-limb over the course of the study. One dog (dog 2; see Figure 1) did not improve with treatment with APS. The cause for this is unclear as there were no apparent difference based upon the CBC data for either whole blood or APS between this dog and the other dogs. Overall, the data demonstrate that at day 28 the APS-treated limbs bore 1.5 units more weight than the saline-treated limbs. Given that each pelvic limb bore ~20 units of body weight at the beginning of the study, a difference of 1.5 between the two limbs represent a relative difference of about 7.5%. These results are believable and consistent with a prior study in dogs with elbow or stifle OA that demonstrated superiority of APS to saline control using objective kinetic data derived from a force plate.

Three out of the 5 assessments performed with the CBPI and LOAD questionnaires showed that owners believed there were statistically significantly improvements over the 28-day study. The two remaining assessments using the CBPI and LOAD trended toward statistical significance with *p*-values <0.1. The consistency of results among the objective kinetic data and the owner questionnaires could be interpreted as supporting the conclusion that the APS was beneficial. However, interpreting data from these questionnaires is limited because each dog was treated with both APS and a saline control and neither the CBPI or LOAD discriminate among limbs. Rather, these questionnaires assess the owner's impression of the dog's function, rather than that of an individual limb. While recognizing this limitation prior to conducting the study, we elected to collect these data because we believed having an owner assessment of function was potentially valuable. However, because of this aforementioned limitation, because owner assessments are subjective, and can be prone to caregiver placebo effect (3), and because objective kinetic data are considered the gold standard outcome measure in treatments for osteoarthritis, we selected the objective kinetic data as the primary outcome measure *a priori*.

An additional implicit objective of the study was to assess safety of the APS treatments. Two patients had mild and self-limiting adverse events that may, or may not have, been related to the use of the APS. Neither patient received treatment and both had resolution of the pruritus or soreness within 72 h following injection. Given that previous studies on APS in horses and dogs showed no adverse events in any animals, it is believable that no serious adverse events were observed in the current study. In turn, we conclude that APS treatment is safe.

There are a few limitations of the current study. First, this study was designed to evaluate the relative benefits of two different treatments in dogs that were symmetrical in terms of their lameness and using relative pressure distribution as the primary outcome measure. The dogs were subjectively symmetrical and their relative pressure distribution was not statistically significantly different between limbs at the start of the study (*p* = 0.49). However, the limbs were not absolutely identical. Figure 1 shows that the relative pressure distribution was greater on the limb that was subsequently treated with APS in 3 of 5 patients and the saline-treated limbs bore more weight at the start of the study in 2 of 5 patients. Therefore, it is possible that the APS treated limbs were less severely affected and more likely to improve. With that potential limitation stated, we don't think that is a relevant concern that renders the data invalid. Overall the dogs were quite symmetrical (Table 2) and we think that a distribution of 3 of 5 dogs having a higher initial TPI on the APS-treated limb vs. 2 of 5 dogs having a higher initial TPI on the saline-treated limb demonstrates that the randomization worked adequately in this study.

The greatest limitation of this study was that the pressure mat used did not provide an absolute value of either peak vertical force or vertical impulse. Rather, the mat provides a relative pressure distribution of each of the dogs' four limbs. As a result, it is not possible to say whether the absolute value of weight bearing (i.e., peak vertical force or vertical impulse) on each limb increased or decreased. For example, it appears that weight bearing on the saline-treated limb decreased and that weight bearing on the APS-treated limbs increased, albeit not significantly. Indeed, it is possible that had we obtained data on the peak vertical force or vertical impulse that these values would have decreased over time in the saline-treated limbs while just remaining constant for the APS-treated limbs. This is feasible because three patients were receiving NSAIDs prior to enrollment in the study. Discontinuation of such medication 7 days prior to study enrollment may have precipitated deterioration in function for the saline-treated limbs. However, it is also feasible that weight bearing (peak vertical force or vertical impulse) in the saline treated-limbs remained constant while weight bearing in the APS-treated limbs increased. If peak vertical force and vertical impulse remained constant in the saline-treated limbs, while increasing in the APS-treated limbs, there would be a relative decrease in pressure distribution to the saline-treated limbs and a relative increase in pressure distribution to the APS-treated limbs, as was documented in this study. We cannot definitively differentiate between these two potential explanations for the data obtained. We can only conclude that the APS treated limbs bore significantly more weight than the control group at day 28 and that the increase in relative weight distribution was significantly greater for the APS-treated limbs than the saline treated limbs.

In conclusion, this pilot study was adequately powered to demonstrate that APS is superior to saline (sham) control for the treatment of hip osteoarthritis in dogs. In turn, although one objective of this study was to perform a power analysis to determine how many dogs would need to be enrolled in a similarly conducted study to detect statistical significance of APS when compared to saline, clearly no power analysis, or further study were needed given that statistical significance was reached in this pilot study. The study likely achieved statistical significance with few dogs because dogs with bilateral disease were included allowing one limb to be used as a treatment limb and one limb to be used as a control. In turn, inter-dog variability was controlled for, thus increasing sensitivity for detecting a statistically significant difference. However, there remain several clinically relevant questions that remain such as whether APS treatment is superior to other clinically relevant treatments and what is the duration of benefit of APS. Given three studies have now demonstrated a proof of principle regarding the efficacy of APS when compared to saline over short time frames (1–3 months), future study should focus on addressing the two aforementioned and currently unanswered questions regarding the efficacy of APS when compared to other clinically relevant treatments and the duration of APS benefit in dogs with OA.

## Data Availability

The datasets generated for this study are available on request to the corresponding author.

## Ethics Statement

The study was reviewed and approved by the Animal Care and Use Committee of Colorado Canine Orthopedics and Rehab.

## Author Contributions

SF designed the study, executed data collection, performed some of the statistical analyses, and wrote the manuscript.

### Conflict of Interest Statement

The author declares that this study received funding from Owl Manor. The funder was not involved in data collection, analysis, interpretation, or the writing of this article or the decision to submit it for publication. The funder was involved in reviewing and approving the study design prior to deciding to fund the study. SF is also a consultant for Arthrex Vet Systems and has received research support from Arthrex Vet Systems and MediVet Biologics to evaluate orthobiologics.
